# Quantitative analysis of the compliance of EU Sewage Sludge Directive by using the heavy metal concentrations from LUCAS topsoil database

**DOI:** 10.1007/s11356-024-31835-y

**Published:** 2024-01-16

**Authors:** Felipe Yunta, Calogero Schillaci, Panos Panagos, Elise Van Eynde, Piotr Wojda, Arwyn Jones

**Affiliations:** European Commission, Joint Research Centre (JRC), Ispra, VA Italy

**Keywords:** Sewage sludge, Soil pollution, LUCAS soil survey, Heavy metals, Contamination index

## Abstract

In the European Union (EU), a common understanding of the potential harmful effect of sewage sludge (SS) on the environment is regulated by the Sewage Sludge Directive 86/278/EEC (SSD). Limit values (LVs) for concentrations of heavy metals in soil are listed in Impact Assessment of this directive, and they were transposed by EU member states using different criteria. Member states adopted either single limit values or based on soil factors such as pH and texture to define the maximum limit values for concentrations of heavy metals in soils. Our work presents the first quantitative analysis of the SSD at the European level by using the Land Use and Coverage Area Frame Survey (LUCAS) 2009 topsoil database. The reference values at the European level were arranged taking into account the upper value (EU_UL) and the lower value (EU_LL) for each heavy metal (arsenic, cadmium, copper, chromium, mercury, nickel, lead, and zinc) as well as taking into account the pH of the soil (cadmium, copper, mercury, nickel, lead, and zinc) as introduced in the SSD Annex IA. Single and integrated contamination rate indices were developed to identify those agricultural soils that exceeded the reference values for each heavy metal. In total, 10%, 36%, and 19% of the LUCAS 2009 topsoil samples exceeded the limit values. Additionally, 12% and 16% of agricultural soils exceeded the concentration of at least one single heavy metal when European LVs were fixed following the soil pH in Strategy II compared to those national ones in Strategy I. Generally, all member states apply similar or stricter limit values than those laid down in the SSD. Our work indicates that choosing LVs quantitatively affects further actions such as monitoring and remediation of contaminated soils. The actual soil parameters, such as heavy metal concentrations and soil pH values from the LUCAS 2009 topsoil database, could be used by SSD-involved policy stakeholders not only to lay down the LVs for concentrations of heavy metal in soils but also for monitoring the SSD compliance grade by using the LUCAS surveys over time (past and upcoming LUCAS datasets).

**Supplementary Information:**

The online version contains supplementary material available at 10.1007/s11356-024-31835-y.

## Introductions

Sewage sludge (SS) is the by-product of domestic and urban wastewater treatment plants, septic tanks, and similar sewage treatment plants. In the European Union (EU), the SS Directive has regulated the potentially harmful effect of SS on the environment and human health (Council Directive 86/278/EEC of 12 June 1986) with the common understanding and field data available at the time. The SS Directive (SSD), with its amendments and updates, regulates the use of SS in agricultural land and sets rules on the type of treatment and sites where SS is used, how farmers can use SS as a fertilizer and soil improver, on the regular recording and reporting of the sludge quantities produced and applied. In 2018, Decision (EU) 2018/853 amended the SSD regarding procedural rules in environmental reporting. In 2019, the SSD was amended by Regulation (EU) 2019/1010, which aligns and streamlines the reporting requirements in environmental legislation. The SSD aims to increase SS use in agriculture while preventing harm to the environment and human health by ensuring that heavy metals in soil and sludge do not exceed set limits (Collivignarelli et al. 2019).

In terms of heavy metals, the list of annexes in the SSD reports the specific LVs (LVs) of heavy metals (HMs) in the soil (Annex IA), maximum concentrations of HMs in sludge (Annex IB), and maximum annual quantities of HMs that may be added to the soil (Annex IC).

Article 5 of the SSD states that Member States should ban the spreading where the concentration of one or more heavy metals in the soil exceeds the LVs which are mentioned in Annex IA (Sewage Sludge Directive). To this end, the SSD includes seven metals (cadmium, copper, chromium, mercury, nickel, lead, and zinc) that, in certain conditions, may be toxic to plants and humans. Directive 86/278/EEC (Annex IA) states that the LVs for Cr could not be fixed because of insufficient scientific data, and the Council would appoint these LVs later. The SSD consequently bans the use of SS, which results in concentrations of these HMs in soil exceeding these LVs. Therefore, identifying sites exceeding the LVs laid down in the SSD is a pivotal factor to consider before applying SS to agricultural land.

Analysis of European Sewage Sludge Normative implementation at the national level has been discussed elsewhere (Bauer et al. 2020; Mininni et al. 2015; Collivignarelli et al. 2019), but only taking into consideration the limit values as laid down in the corresponding directives. However, no quantitative analysis by using real experimental soil data at the European level has been already done. Some national guidelines use soil properties such as soil pH and texture to define the LVs of concentrations of HM in soils (Braguglia et al. 2015; Gianico et al. 2021; Kacprzak et al. 2017). The Sewage Sludge Directive allows laying down the LVs for concentrations of heavy metals in soils per soil pH (Annex IA of SSD), but soil texture has also been implemented for some Member States. Further investigations should be conducted to analyze how soil parameters are used for the implementation of LVs by using the actual content of heavy metals in the soils of Europe. Application of SS on agricultural soils will depend on the concentration of heavy metals in both SS and soil. The average concentration of heavy metals from 61 sludges sampled along Europe was reported by Tavazzi et al. (2012). Still, the potential impact of their application on agricultural land still needed to be done as no concentrations of heavy metals in soils were taken into consideration. Huygens et al. (2022) suggested potential risks for human health and the environment from certain persistent, bioaccumulative, and toxic organic compounds when sludge was applied over time on agricultural land, but no specific analysis on heavy metals was performed. It should also be done for metals, as no risk assessment studies have yet been conducted. This work aims to develop a methodology which contributes to the analysis of the implementation grade of the EU Sewage Sludge Directive by using the actual concentration of heavy metals in agricultural soils of Europe from the Land Use/Cover Area Frame Statistical Survey (LUCAS) 2009 topsoil survey (LUCAS 2017). Sites exceeding LVs were first identified to estimate the proportion of agricultural points where sludge cannot be spread. Then, the level of SSD implementation in Europe was determined by comparing the LVs laid down by the member states with those laid down by the SSD.

## Material and methods

### LUCAS and soil samples analysis

#### LUCAS topsoil database

The Land Use/Land Cover Area Frame Survey (LUCAS) is a European field survey program to monitor land use and cover changes in the European Union. Three LUCAS surveys have been completed in 2009, 2015, and 2018, and the latest is ongoing (2022). Every composite soil sample results from collecting five topsoils (0–20 cm) subsamples per location. Approximately 500 g of soil were air-dried and transferred to the core laboratory to measure physical (particle size distribution, coarse fragments, etc.) and chemical properties (pH, carbonates, soil organic carbon, nitrogen, phosphorus, potassium, etc.) by using standardized laboratory methods (Panagos et al. 2021; Palmieri et al. 2011; Orgiazzi et al. 2018, 2022). In 2009, the LUCAS survey included a dedicated module for soil pollution monitoring. Metals and metalloids such as arsenic (As), cadmium (Cd), cobalt (Co), chromium (Cr), copper (Cu), mercury (Hg), nickel (Ni), lead (Pb), antimony (Sb), vanadium (V), and zinc (Zn) were included.

#### Soil samples analysis

LUCAS Topsoil Database 2009 collected 14,726 points placed in agricultural lands (croplands and grasslands as identified by the surveyors at the sampling time) for EU countries (excepting Croatia) and the UK (Land Use/Cover Area frame Statistical Survey-LUCAS, 2017). Pseudototal concentrations of arsenic (As), cadmium (Cd), chromium (Cr), copper (Cu), mercury (Hg), nickel (Ni), lead (Pb), and zinc (Zn) were measured by using Inductively Coupled Plasma Optical Emission Spectrometry (ICP-OES) after acid digestion with aqua regia (ISO 11466: 1996) and microwave-assisted acid digestion (European Committee for Standardization EN 16174:2012). Mercury was not determined in the 91 soil samples from Cyprus, but the rest of the metals were taken into consideration. The limit of quantification (LOQ) for As, Cd, Cr, Cu, Hg, Ni, Pb, and Zn were determined to be 2.84, 0.07, 0.32, 0.26, 0.054, 0.27, 1.16, and 2.12 mg kg^−1^, respectively (Tóth et al. 2016 and Panagos et al. 2021). Soil samples with concentrations below the limit of quantification (LOQ) were recalculated to have a value equal to half of the quantification limit as already used elsewhere (Tóth et al. 2016, Panagos et al. 2018, Hornung and Reed 1990, Nehls and Akland 1973). Analysis and determination of the soil parameters such as pH and texture were performed by following the corresponding ISO standard methods reported by Tóth et al. (2013a) as they were later used as soil criteria for fixing LVs.

### Use of limit values to assess metal contamination

Three strategies were managed in this work according to the diverse ways as LVs for heavy metal concentrations in soil laid down in the current SSD are transposed by the Member States and the UK (Table 1). Concentrations of measured heavy metals from the (LUCAS) 2009 database were compared to the LVs for each proposed strategy. The strategy we are proposing tries to fix the measured heavy metal concentrations (As, Cd, Cu, Cr, Hg, Ni, Pb, and Zn) to both the upper (EU_UL) and lower (EU_LL) LVs separately as laid down in the SSD (Annex IA of Directive 86/278/EEC). The LVs of As and Cr were estimated at 25.5 and 100 mg kg^−1^, respectively, as the average values available by the Member States legislations. It was done to enable a complete spatial analysis of the results and to compare them to the national LVs. Strategy II was designed to fix the measured heavy metal concentrations (Cd, Cu, Hg, Ni, Pb, and Zn) to the LVs for each heavy metal following the experimental soil pH (Annex IA of Directive 86/278/EEC) for each soil sample from LUCAS 2009 database. Three LV levels were defined along pH interval (i.e., lower LVs for acid soils with pH below 6, intermediate LVs for neutral soils with pH between 6 and 7, and higher LVs for alkaline soils with pH above 7). The As and Cr were not considered in strategy II as no LVs at different soil pH values have been set. Strategy III took into consideration the national LVs as laid down by each Member State in their national legislations and summarized by Mininni et al. (2015), Collivignarelli et al. (2019), and Bauer et al. (2020).

**Table 1:** Limit values for concentrations of HMs in agricultural soils (mg kg^−1^ dry matter) used for the Strategies I, II, and III, respectively

		As	Cd	Cr	Cu	Ni	Pb	Zn	Hg
Strategy I (fixed)									
Directive 86/278/EEC	EU_LL	25.5^1^	1.0	100^1^	50	30	50	150	1.0
	EU_UL	25.5^1^	3.0	100^1^	140	75	300	300	1.5
Strategy II (pH dependent)									
Directive 86/278/EEC	6 ≥ pH	-	1.0	-	50	30	50	150	1.0
	6 < pH ≤ 7	-	3.0	-	140	75	300	300	1.5
	pH > 7	-	4.5	-	210	112	450	450	2.25
Strategy III (national)									
Austria (rest of the regions)		25.5^1^	1.4^1^	91^1^	81^1^	55^1^	90^1^	231^1^	0.9^1^
Lower Austria		25.5^1^	1.5	100	60	50	100	200	1.0
Upper Austria		25.5^1^	1.0	100	100	60	100	300	1.0
Burgenland		25.5^1^	2.0	100	100	60	100	300	1.5
Vorarlberg		25.5^1^	2.0	100	100	60	100	300	1.0
Steiermark		20.0	2.0	100	100	60	100	300	1.0
Carinthia									
5.5 ≥ pH		25.5^1^	0.5	50	40	30	50	100	0.2
5.5 < pH ≤ 6.5		25.5^1^	1.0	75	50	50	70	150	0.5
pH > 6.5		25.5^1^	1.5	100	100	70	100	200	1.0
Belgium									
Flanders		22.0	0.9	46	49	18	56	162	1.3
Walloon		22.0	2.0	100	50	50	100	200	1.0
Bulgaria									
7.4 ≥ pH		25.0	2.0	200	100	60	80	250	1.0
pH > 7.4		25.0	3.0	200	140	75	100	300	1.0
Cyprus		25.5^1^	3.0	100^1^	140	75	300	300	1.5
Czech Republic									
Common soils		20.0	5.0	90	60	50	60	120	0.3
Sands, clay sands, gravel sands		15.0	4.0	55	45	45	55	105	0.3
Denmark		25.5^1^	0.5	30	40	15	40	100	0.5
Finland		25.5^1^	0.5	200	100	60	60	150	0.2
France		25.5^1^	2.0	150	100	50	100	300	1.0
Germany									
Clay		25.5^1^	1.5	100	60	70	100	200	1.0
Loam/silt		25.5^1^	1.0	60	40	50	70	130^1^	0.5
Sand		25.5^1^	0.4	30	20	15	40	60	0.1
Greece		25.5^1^	3.0	100^1^	140	75	300	300	1.5
Ireland		25.5^1^	1.0	100^1^	50	30	50	150	1.0
Italy		25.5^1^	1.5	100^1^	100	75	100	300	1.0
Luxembourg		25.5^1^	3.0	200	140	75	300	300	1.5
Estonia		25.5^1^	3.0	100	50	50	100	300	1.5
Hungary		25.5^1^	1.0	75	75	40	100	200	0.5
Latvia		25.5^1^	0.9	90	70	70	40	100	0.5
Lithuania		25.5^1^	1.5	80	80	60	80	260	1.0
Malta									
6 ≥ pH		25.5^1^	0.5	30	20	15	70	60	0.1
6 < pH ≤ 7		25.5^1^	1.0	60	50	50	70	150	0.5
pH > 7		25.5^1^	1.5	100	100	70	100	200	1.0
Netherlands^2^		20.0	0.6	55	40	35	50	140	0.3
Portugal									
5.5 ≥ pH		25.5^1^	1.0	50	50	30	50	150	1.0
5.5 < pH ≤ 7		25.5^1^	3.0	200	100	75	300	300	1.5
pH > 7		25.5^1^	4.0	300	200	110	450	450	2.0
Poland									
Light soil		25.5^1^	1.0	50	25	20	40	80	0.8
Medium soil		25.5^1^	2.0	75	50	35	60	120	1.2
Heavy soil		25.5^1^	3.0	100	75	50	80	180	1.5
Romania		25.5^1^	3.0	100	100	50	50	300	1.0
Slovakia		25.0	1.0	60	50	50	70	150	0.5
Slovenia									
6 ≥ pH		25.5^1^	1.0	100	60	50	85	200	0.8
pH > 6		25.5^1^	1.0	30	20	15	70	60	0.8
Spain									
7 ≥ pH		25.5^1^	1.0	100	50	30	50	150	1.0
pH > 7		25.5^1^	3.0	150	210	112	300	450	1.5
Sweden		25.5^1^	0.4	60	40	30	40	100	0.3
UK		50.0	3.0	400	200	110	300	450	1.0

^1^Data not found. Average values were used for the analysis

^2^LVs for the Netherlands were taken from the national SS legislation

Strategy III considers the LVs laid down for each Member State and the soil parameters used as screening criteria (e.g., pH and texture). A methodological approach was developed by considering the soil texture requirements of some Member States (Table 2). The LVs for the Czech Republic were classified using two soil textural categories: “common soils” and “sands/clay sands/gravel sands” based on the sand content. Germany’s LVs were defined using three soil texture categories: sand, loam/silt, and clay. Based on clay content, Poland represented them following three soil textural levels (light, medium, and heavy). USDA-based soil texture classes were first calculated by combining the clay, silt, and sand percentages experimentally obtained from LUCAS 2009 soil samples (Tóth et al. 2013a). The twelve resulting USDA-based soil texture classes were merged correctly following the textural requirements of Germany, Poland, and the Czech Republic, as shown in Table 2. The concentration for each heavy metal of the LUCAS 2009 topsoil database was compared with its reference LV using the new texture classes. The stricter LVs of HMs were found for soils with lower clay content due to lower soil adsorption capacity, increasing heavy metal availability in soils. Soils with high cation exchange capacity would have a greater retention capacity for cationic HMs, and, therefore, their bioavailability in soils decreases (Xu et al. 2017). Thus, 48 national and regional LVs were managed in Strategy III compared to Strategies I and II. These calculations were performed by using a Microsoft Office Excel® spreadsheet.

**Table 2:** Classes of soil texture used in the Strategy III to fix the limits values for HMs in the soils (mg kg^−1)^

General texture	USDA Textural class	Sand	Silt	Clay	Germany	Poland	Czech Republic
Sandy soils (coarse texture)	Sand	86–100	0–14	0–10	Sand	Light	Sands, clay sands, gravel sands
	Loamy sand	70–86	0–30	0–15			
Loamy soils (moderately coarse texture)	Sandy loam	45–70	0–50	0–20	Loam/silt	Medium	Common soils
Loamy soils (medium texture)	Loam	23–52	28–50	7–27			
	Silty loam	0–50	50–80	0–27			
	Silt	0–20	80–100	0–12			
Loamy soils (moderately fine texture)	Clay loam	20–45	15–52	27–40			
	Sandy clay loam	45–80	0–28	20–35			
	Silty clay loam	0–20	40–73	27–40			
Clayey soils (fine texture)	Sandy clay	45–65	0–20	35–55	Clay	Heavy	
	Silty clay	0–20	40–60	40–60			
	Clay	0–45	0–40	40–100			

### Methods and data analysis

New contamination rate indices were defined in this work to be used not only to identify the agricultural soil samples exceeding the LVs but also to know the remaining concentration before reaching LVs.

Single Contamination Rate Indices ($${CRI}_{EU}^{i}$$) and $${(CRI}_{Nat}^{i})$$ were determined for each “$$i$$” heavy metal from each LUCAS 2009 agricultural soil sample by dividing the measured concentration ($${C}_{n}^{i} \mathrm{in mg kg}-1$$) by its corresponding LV (mg kg^−1^) as previously defined either at European level ($${C}_{EU}^{i})$$ for strategies I and II or at national level ($${C}_{Nat}^{i}$$) for strategy III (see Eqs. 1 and 2, respectively).

1 $${CRI}_{EU}^{i}=\frac{{C}_{n}^{i}}{{C}_{EU}^{i}}\cdot 100$$

2 $${CRI}_{Nat}^{i}=\frac{{C}_{n}^{i}}{{C}_{Nat}^{i}}\cdot 100$$

where $$i=As, Cd, Cu, Cr, Hg, Ni, Pb,\, and\, Zn\, when\, comparing\, strategies\, I\, and\, III$$

$$i=Cd, Cu, Hg, Ni, Pb,\, and\, Zn\, when\, comparing\, strategies\, II\, and\, III$$

Overall Contamination Rate Indices, $O{{\text{CRI}}}_{{\text{EU}}}$ and $O{{\text{CRI}}}_{{\text{Nat}}} ,$ were determined by taking the highest value from (${CRI}_{EU}^{i}$) and${(CRI}_{Nat}^{i})$, respectively (see Eqs. 3 and 4), for each LUCAS 2009 soil sample. Hence, in accordance with the “One-Out-All-Out” approach, a site will exceed the LVs as soon as the concentration of a single HM exceeds its reference LV. The “one-out, all-out” principle has been already proposed to be used when defining soil health as it implies that a soil would be classified as not healthy when at least one of the indicator thresholds is not met (Bouma and Veerman 2022). Contamination rates above 100 were normalized to be 100 as these soil samples exceed the limit values and no sludge should be spread.

3 $$O{{\text{CRI}}}_{{\text{EU}}}={\text{MAX}}\left[{{\text{CRI}}}_{{\text{EU}}}^{{\text{i}}}\right]$$

4 $$O{{\text{CRI}}}_{{\text{Nat}}}={\text{MAX}}\left[{{\text{CRI}}}_{{\text{Nat}}}^{{\text{i}}}\right]$$

Distribution of Contamination Rate Indices was aggregated to country (NUTS0) and regional (NUTS2) levels by calculating the arithmetic average values using NUTS2006 Eurostat administrative limits (Regulation (EC) No 1059/2003).

Analysis of the grade of compliance of SSD was calculated quantitatively as the ratio between OCRINat and OCRIEU values for each LUCAS 2009 soil sample by using those data from strategies III and II, respectively. Ratios lower than 0.80 indicate that European LVs are stricter than national ones. Conversely, ratios above 1.30 indicate that those LVs adopted by the Member States are more stringent than those established in the SSD. A good balance between both LVs was defined for ratios between 0.8 and 1.3, as around 25–30% deviations were considered acceptable.

## Results

### Overall heavy metal exceedance rate index in EU agricultural soils

**Fig. 1:**
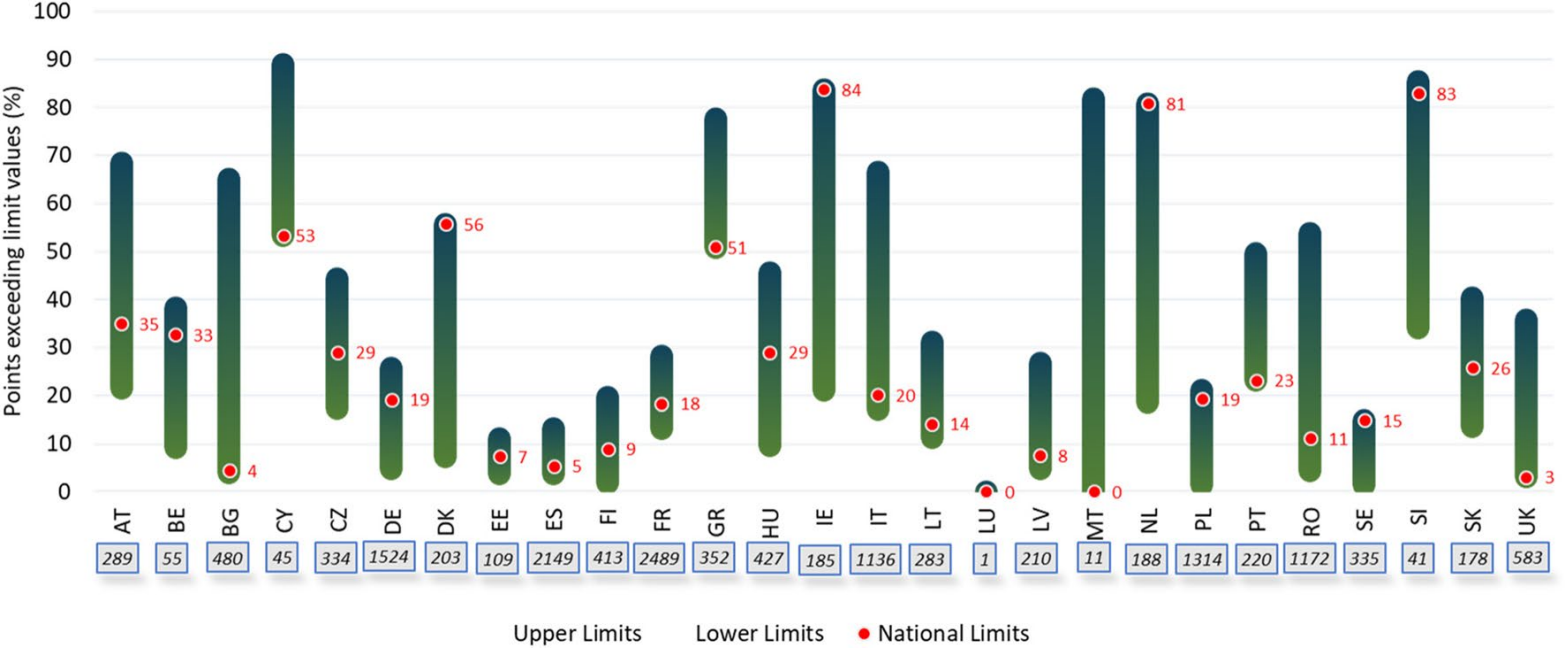
Distribution of soil samples (in percentage) exceeding the EU LVs (lower EU LVs correspond to the bar top and upper EU LVs correspond to the bar bottom) and the national ones (red spots). A number of agricultural soils sampled from the LUCAS 2009 database per country are shown in the boxes

The distribution of soil samples exceeding the EU and national LVs from Strategies I and III, respectively, are shown in Fig. 1. A similar proportion of soil samples exceeding LVs were found for Denmark, Ireland, the Netherlands, Slovenia, Poland, Sweden, and Belgium when using national and lower EU LVs. Conversely, countries such as Bulgaria, Cyprus, Spain, Greece, Italy, Latvia, Malta, and Portugal transposed those EU upper ones as national LVs. The quantification of points exceeding the LVs varies mainly depending on those adopted. The proportion of soil samples exceeding LVs has a large variability in Austria (22–69%), Bulgaria (4–65%), Ireland (21–84%) Italy (17–67%), the Netherlands (19–46%), Romania (4.3–54%), and Slovenia (34–86%) when EU upper or lower LVs were compared, respectively. This difference was extreme in the case of Malta since soil samples exceeding the LVs ranged from 0 to 82% when upper or lower LVs were compared, respectively. This finding should be managed as preliminary, as only 11 soil samples were analyzed in Malta for this work. Still, it clearly shows the diverse countability following the LVs used. Although the transposition of EU LVs by the Member States was adopted in various ways, all national LVs fall into the EU LVs interval, indicating that all of them comply with the SSD LVs.

**Fig. 2:**
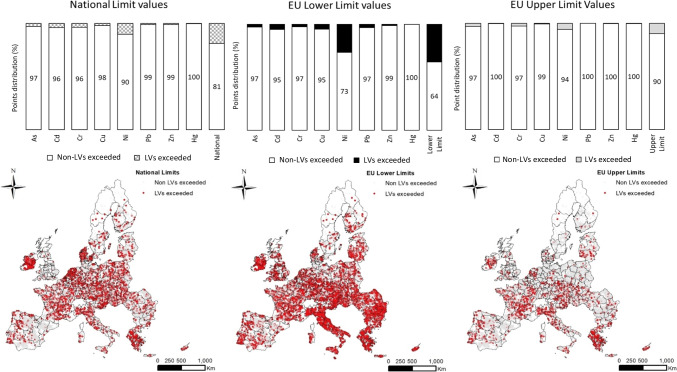
Proportion and spatial distribution of LUCAS 2009 agricultural soils by using the LVs defined in strategy I (maps and bar graphs middle- and right-hand) and strategy III (map and bar graph left-hand)

Proportions of soil samples exceeding LVs for each heavy metal (As, Cd, Cu, Cr, Hg, Ni, Pb, and Zn) as well as their spatial distribution are shown in Fig. 2 when both EU and national LVs were compared following the Strategies I and III, respectively. Ten percent of soil samples exceeded the LVs when using the EU-Upper ones (EU_UL) (Fig. 2, right side). The proportion of sites exceeding LVs was lower than 6% for all tested heavy metals (As, Cd, Cr, Cu, Hg, Ni, Pb, and Zn). They are mostly placed in Greece, Northern Italy (Piemonte, Val d’Aosta, and Emilia-Romagna), and Ireland. However, 36% of LUCAS 2009 agricultural soils exceeded the LVs when lower LVs (EU_LL) were analyzed. These soils were placed in Romania, Bulgaria, and Italy (Central and South). Using the EU_LL LVs, countries such as Ireland, Italy, Greece, Cyprus, Malta, and some regions in southern Portugal, Bulgaria, and Romania were identified as areas where further analysis should be carried out before SS application (Fig. 2, middle) as a substantial number of soil samples exceed the LVs. Finally, 19% of agricultural soil samples from the LUCAS 2009 topsoil database exceed the LVs when using national LVs (Fig. 2, left side). Countries such as Ireland and Denmark show a similar distribution of soil samples exceeding the LVs when comparing the EU-LL or national ones.

### Heavy metals that limit SS application

A comparison of soil samples exceeding the LVs following strategies I and III for each heavy metal is shown in the supplementary material (Fig. 1S). Some soil samples from Greece showed an exceedance regardless of the LVs tested. This indicates that these soils showed an elevated concentration of heavy metals. Specifically, Cr and Ni (Fig. 1S) and, therefore, the application of SS has to be prevented here. Ireland also shows high levels of Ni and Cd (Fig. 1S). When using EU_LL values, we identified soils exceeding LVs in Romania, Bulgaria, and Italy (Central and South). For these countries, Ni seems to be the heavy metal hampering the potential application of SS, as 27% of soils show concentrations higher than the lower EU LVs of 30 mg kg^−1^ (Fig. 2, middle bar graph). The SSD does not include LVs for Cr and As, so for the analyses in this study, we used the average of national LVs across member states for these two metals.

Soils exceeding the tested Cr LVs were placed mainly in Greece and northern Italy (Piemonte and Emilia-Romagna). Only 3% of agricultural soils from the LUCAS 2009 database exceeded the As LVs when using those proposed LVs. It does not seem that As limits the application rate of SS in European agricultural soils when 25.5 mg kg^−1^ was used. However, the few sites exceeding the As thresholds are concentrated in some regions in France. Since As is carcinogenic to humans (Mass 1992), the occurrence of these limited sites exceeding LVs still deserves further investigation to assess the origin of As in these soils. Lead affects some regions in the UK, Italy, and Austria when lower LVs are used in the analysis. Copper contamination rate was detected in some areas of Italy, south of France and Greece when lower LVs were used (Fig. 1S). Some significant differences were detected between national and European LVs for mercury. National LVs for the Czech Republic, Finland, Germany, the Netherlands, and Sweden are 50% lower than those European lower LVs, although no soil samples exceeding the LVs were found. Since background information on national LVs is often missing, the large discrepancies for mercury ask for further investigation to find a reliable limit value for mercury.

To conclude, we found that SS application across Europe is mainly limited by Ni, followed by Cr and Cu, with differences between member states.

### Spatial assessment of heavy metal contamination in Europe

**Fig. 3:**
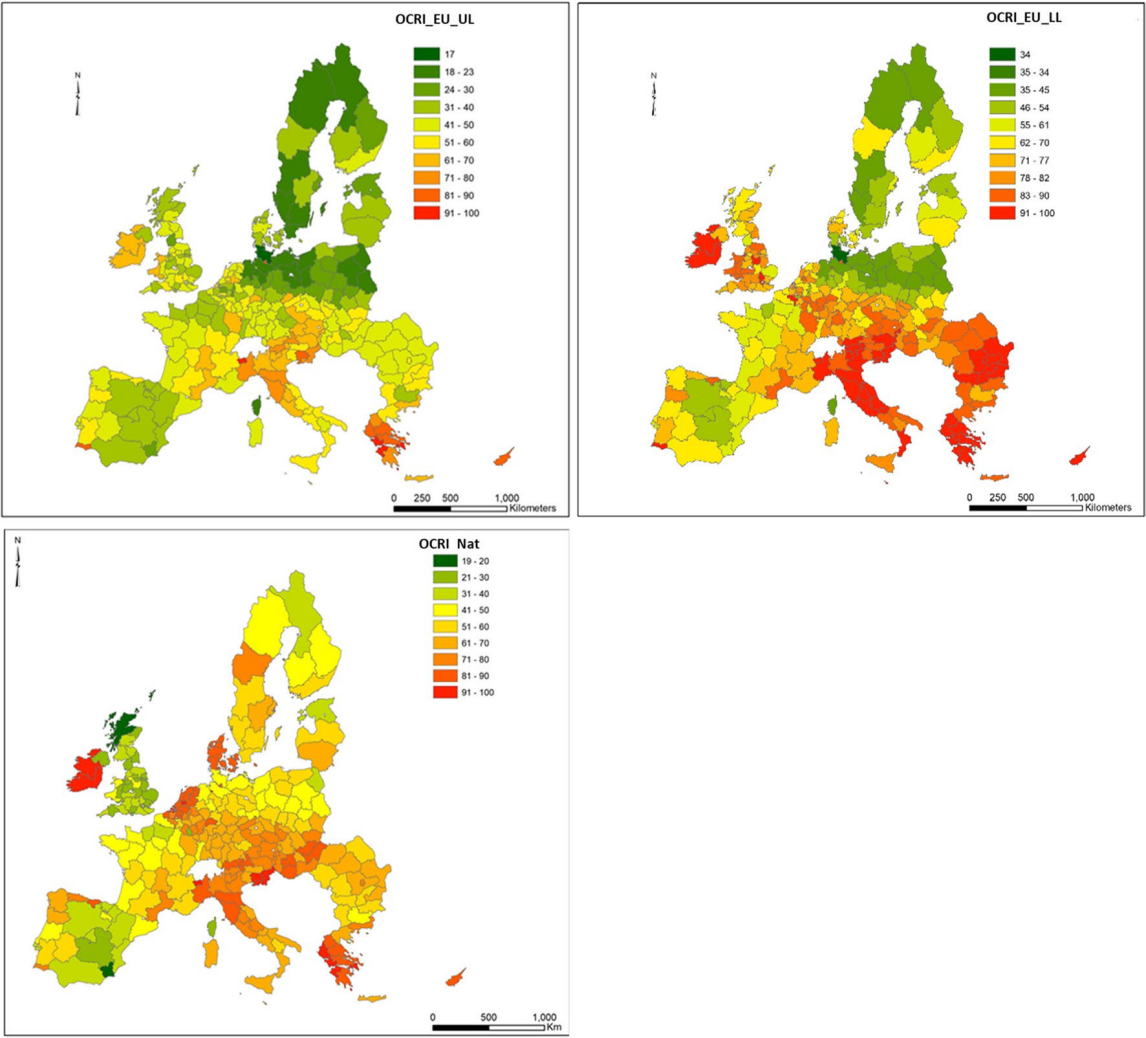
Distribution of the average OCRI values aggregated at NUTS2 level from LUCAS 2009 database by using the LVs defined in strategy I (top maps) and strategy III (bottom map)

**Fig. 4:**
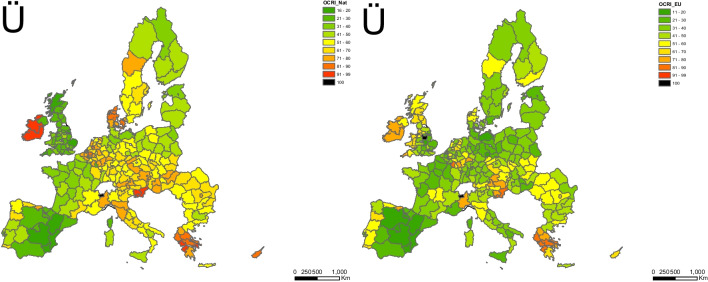
Distribution of the average OCRI values aggregated at NUTS2 level from the LUCAS 2009 database by using the LVs defined in strategy II (right-hand map) and strategy III (left-hand map)

The spatial distribution of average OCRI values aggregated at the NUTS2 level is shown in Fig. 3 when using the LVs from strategies I and II (Table 1). Single CRI values for each heavy metal aggregated at the NUTS2 level are shown in Figs. 2S, 3S, and 4S when national, EU_LL, and EU_UL levels were used, respectively.

Greece and Cyprus show OCRI values above 80% for both strategies. Ireland, Denmark, the Netherlands, Greece, and Slovenia resulted in overall contamination rates above 80% when strategy III was transposed (Fig. 2, lower map). Large overall contamination rates for Denmark and the Netherlands based on national LVs can be explained by the stricter LVs at the national level, as they are lower than the low EU LVs from the SSD. Regions in the north of Italy (such as Val d’Aosta, Piemonte, Emilia Romagna, and Trento) and southern Portugal (Algarve) should be carefully analyzed before SS application as they show OCRI values above 70% based on upper EU-LVs (EU_UL) (top left map in Fig. 3). Agricultural soils from Ireland, Italy, Slovenia, Austria, Romania, Bulgaria, Greece, Cyprus, and Malta show contamination rates above 70% based on lower limit European values (EU_LL) (top right map in Fig. 3). As already introduced, countries such as Denmark, the Netherlands, Belgium, Sweden, and some regions from Germany show significantly lower national LVs than those lower ones laid down in the SSD, and it predominantly affects the OCRI values higher than 70%. As introduced in Fig. 1, the OCRI indices could vary depending on the LVs adopted. The contamination rates for the Netherlands could decrease from 85 to 50–70% if the lower and the upper LVs from SSD were adopted, respectively. In the case of Sweden, the current OCRI is around 50%, and it could be lowered to 20% if those upper LVs from strategy I were applied. Conversely, the overall contamination rate in the UK is around 25% based on national legislation limits. These scenarios could be increased to reach up to 70% (on average) if lower LVs from the strategy I were adopted. Therefore, the determination of contamination rate indices in soils seems to be a convenient methodological approach not only to identify sites exceeding the LVs but also to detect warning areas where the application of sludge could lead to exceed the LVs as highest contamination rates were found in some Italy, Greece, Slovenia, Germany, and Ireland areas.

### The effect of using soil-specific limit values

**Fig. 5:**
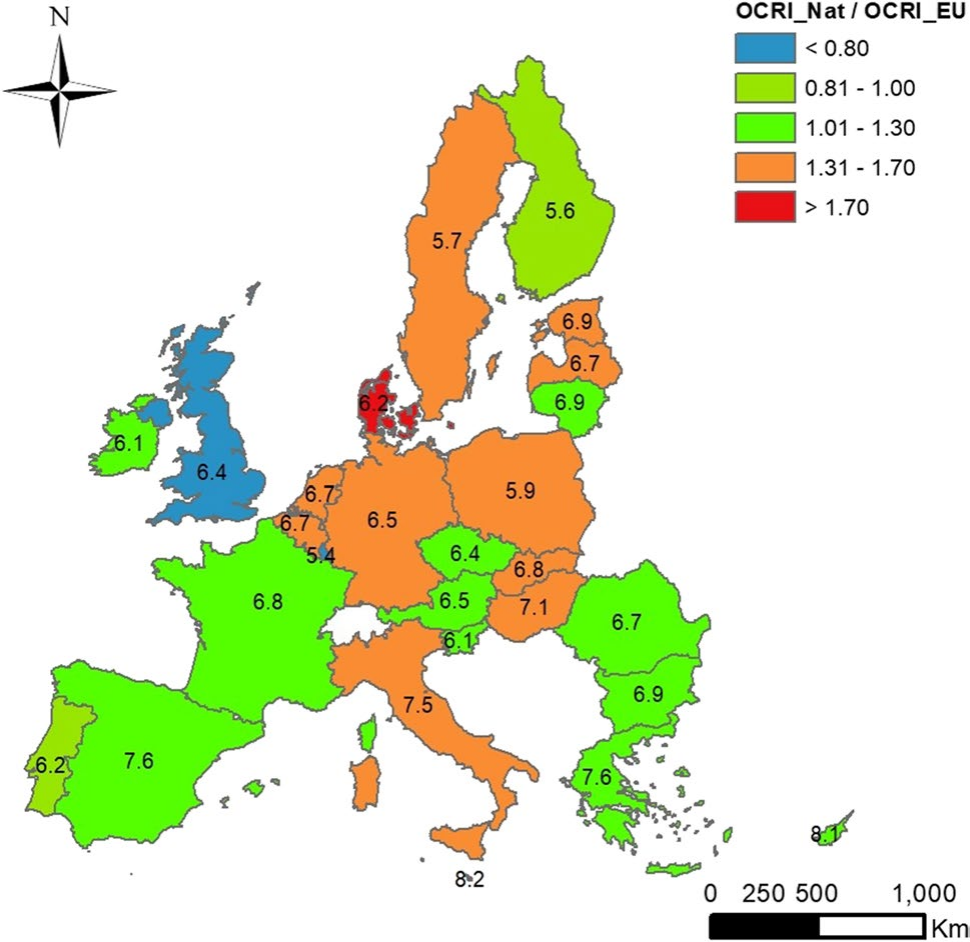
Distribution of the average values of overall grade of compliance aggregated by country. It was quantitatively calculated as the OCRI_Nat/OCRI_EU ratio between values for each LUCAS 2009 soil sample by using those data from strategies III and II, respectively. Ratios lower than 0.80 indicate that European LVs are stricter than national ones. Ratios above 1.30 indicate that those LVs adopted by the Member States are more stringent than those established in the SSD. Values ranged from 0.8 to 1.3 are indicating a good compliance grade. Mean values of soil pH (in water) were included per country

Figure 4 shows the average OCRI values distribution using the LVs defined in strategies II and III. This analysis included no reference values for As and Cr because they were already analyzed in strategies I and III, and no new LVs along soil pH were reported. The northern Italy region of Aosta shows an OCRI of 100% regardless of LVs used, indicating that no SS could be applied on agricultural soils, but this finding is no longer representative as only one soil sample was included into the analysis. Member States such as Ireland and Slovenia show OCRI values above 90%, and other ones, such as Greece, Cyprus, and Denmark, higher than 70% when national LVs were adopted. The application of SS in these countries should be assessed case by case and continuously monitored to avoid the exceedance of the LVs. Spatial distribution by using pH-dependent LVs is shown in the supplementary material (Fig. 5S). One or more HM concentrations were exceeded for 12% of agricultural soils when the pH-dependent European LVs were used (Fig. 5S left). The proportion of sites exceeding the LVs for each heavy metal was 1.9% for Cd, 0.9% for Cu, 0.1% for Hg, 8.6% for Ni, 1.0% for Pb, and 0.1% for Zn.

Conversely, total soil samples exceeding LVs were 2367 (corresponding to 16%) and 4.5% for Cd, 2.5% for Cu, 0.9% for Hg, 10.8% for Ni, 1.8% for Pb, and 1.8% for Zn when national LVs were used (Fig. 5S right). Therefore, 676 new agricultural soils exceeding the LVs were identified when national LVs were adopted (national LVs were obtained using Strategy I, excluding data from As and Cr). These significant differences have a profound impact, as 40% of agricultural soils are differently classified depending on the LVs considered. Using national levels allows us to identify new sites exceeding the LVs, mainly in Ireland, Belgium, the Netherlands, Denmark, Sweden, Slovenia, and Cyprus. Conversely, new sites exceeding LVs were identified using the EU LVs in Portugal. These findings align with those previously discussed by using Strategies I and III since stricter LVs at the national level yield higher contamination rates in soils and vice versa. Contamination rate indices allow for the identification of the agricultural soils exceeding the LVs, where no sludge can be applied, as well as the degree of contamination to identify and predict risks in agricultural soils. Further analysis and monitoring activities should be implemented in such land use before applying sludges. Sludge could be applied in all countries since the average OCRI values based on European or national LVs are lower than 100% (Table 3). Single contamination rate indices for each heavy metal are shown in supplementary material (Table 1S).

**Table 3:** Arithmetic average of OCRI values (%) at NUTS0 level (country) by using the LVs (Table 1) defined in Strategies II (OCRI_EU) and III (OCRI_Nat). Standard deviations are shown in parentheses

Country	*N*	OCRI_EU		OCRI_Nat	
Austria	289	61.9	(31.4)	70.5	(27.6)
Belgium	55	41.2	(30.7)	69.9	(26.9)
Bulgaria	480	46.8	(25.3)	53.9	(21.2)
Cyprus	45	66.9	(27.1)	82.0	(24.1)
Czechia	334	52.7	(33.5)	65.7	(26.6)
Germany	1524	33.9	(28.1)	54.4	(28.2)
Denmark	203	44.8	(33.2)	81.8	(24.4)
Estonia	109	24.1	(22.3)	34.0	(23.6)
Spain	2149	19.6	(18.2)	23.3	(22.3)
Finland	413	46.8	(29.3)	44.5	(26.7)
France	2489	34.7	(27.7)	43.4	(28.0)
Greece	352	65.7	(34.1)	72.3	(32.2)
Hungary	427	41.4	(28.9)	68.5	(27.6)
Ireland	185	75.0	(29.1)	95.0	(14.4)
Italy	1136	44.5	(27.5)	59.2	(27.1)
Lithuania	283	34.7	(32.0)	45.0	(31.9)
Luxembourg	1	57.2	-	25.8	-
Latvia	210	30.6	(28.6)	43.7	(26.1)
Malta	11	17.8	(0.0)	31.0	(0.0)
Netherlands	188	48.7	(36.0)	70.3	(30.6)
Poland	1314	34.3	(31.3)	47.0	(32.5)
Portugal	220	55.9	(35.4)	50.1	(34.5)
Romania	1170	49.5	(26.7)	63.5	(21.9)
Sweden	335	36.7	(26.5)	58.7	(27.1)
Slovenia	41	79.8	(24.3)	95.1	(14.2)
Slovakia	178	42.5	(30.5)	59.8	(26.8)
United Kingdom	583	45.1	(29.5)	26.2	(19.8)

### Sewage sludge directive compliance grade

A compliance grade was defined by comparing the OCRI data from Strategy III (no data from As and Cr included) with those of Strategy II. The compliance grade of the SSD is shown in Fig. 5. Member States in green, such as Portugal, France, Ireland, Slovenia, Austria, Czech Republic, Romania, Bulgaria, Greece, Cyprus, Finland, and Lithuania, show similar EU and national LVs. Countries in orange, such as Belgium, the Netherlands, Italy, Germany, Poland, Slovakia, Hungary, Latvia, Estonia, and Sweden, have stricter national LVs than the European ones. Countries in red, such as Denmark and Malta, show the most stringent national LVs, as they are 70% lower than the European ones.

**Fig. 6:**
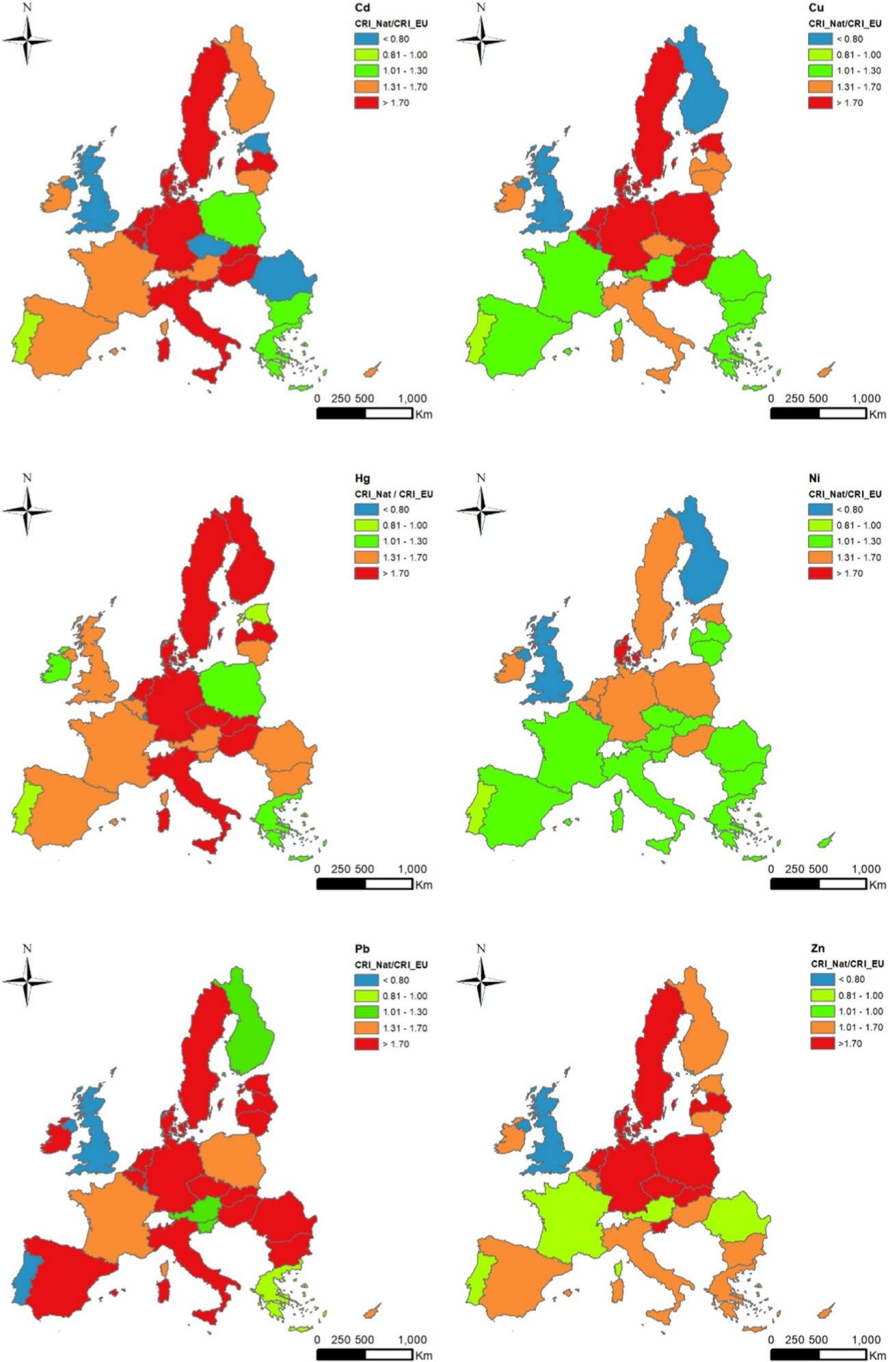
Distribution of the average values of single grade of compliance aggregated by country. It was quantitatively calculated as the OCRI_Nat/OCRI_EU ratio between values for each LUCAS 2009 soil sample by using those data from strategies III and II, respectively. Ratios lower than 0.80 indicate that European LVs are stricter than national ones. Ratios above 1.30 indicate that those LVs adopted by the Member States are more stringent than those established in the SSD. Values ranged from 0.8 to 1.3 are indicating a good compliance grade. Mean values of soil pH (in water) were included per country

Moreover, average soil pH values were shown in the map because this soil factor was used to fix the LVs to explain some inconsistent findings. All countries using soil pH as a screening factor (Portugal, Spain, Slovenia, and Bulgaria) show a high compliance grade, although some of them use different soil pH intervals than those laid down in the SSD. If soil pH values were used as a discriminant factor, countries such as Spain, Italy, Greece, Malta, Cyprus, and Hungary could exceed those upper EU levels by no more than 50% as they have an average soil pH higher than 7. Malta uses the same pH intervals as those laid down by the EU, even though the LVs are stricter than those national ones. Conversely, Finland, Poland, and Sweden could adopt the most stringent EU levels as agricultural soil pH values are lower than six and heavy metal bioavailability is expected to be higher. Comparisons among single contamination rate indices per each heavy metal (Cd, Cu, Hg, Ni, Pb, and Zn) and the number of soil samples per country are shown in Fig. 6. In Finland, it would be advisable to adopt stricter LVs for copper (50 mg kg^−1^) and nickel (30 mg kg^−1^) as average soil pH values are lower than 6.0, and a high heavy metal bioavailability rate is expected. The Czech Republic shows LVs for Cd (4.0 and 5.0 mg kg^−1^) higher than the EU LVs. Cadmium LV of 3.0 mg kg^−1^ should be laid down in the Czech Republic as its average soil pH was 6.4. As already pointed out, the upper LVs for Malta could be increased as it shows the strictest national ones for all heavy metals, even if it has the highest average soil pH (8.2). Greece appears green for all HMs, although no soil pH values were used as a screening factor. The average soil pH value in Greece is 7.6; therefore, the highest LVs from strategy II could be adopted. The LVs for lead in Portugal should be matched to the soil pH since it is 6.2; therefore, LVs of 300 mg kg^−1^ should be laid down. In general terms, most European countries are complying with the SSD as they show equal or stricter national LVs than European ones for all heavy metals. Using single Contamination Rate (CRI) and integrated Overall Contamination Rate (OCRI) indices seems a valuable method to assess the grade of compliance of SSD by using the different LVs. The actual soil parameters, such as heavy metal concentrations and soil pH values from the LUCAS 2009 database, could be used by SSD-involved policy stakeholders not only to lay down the LVs for concentrations of heavy metal in soils but also for monitoring the compliance grade by using the experimental data got from other LUCAS surveys over time (past and upcoming LUCAS datasets).

## Discussion

The Sewage Sludge Directive (86/278/EEC) is currently being revised to promote the application of SS to agricultural lands while ensuring the protection of humans, animals, plants, and the environment. Topics such as LVs for concentrations of heavy metals in soils, maximum concentration of heavy metals in sludge, and maximum quantities of sludge applicable to soil are being revised, and new LVs are usually proposed (Inglezakis et al. 2014). National and EU LVs have already been reviewed by some authors to assess the transposition grade of SSD LVs across Europe (Mininni et al. 2015; Collivignarelli et al. 2019). Additionally, national LVs from Sweden, the Netherlands, and Denmark were also compared to those European ones by Bauer et al. (2020), but no actual measured concentrations of heavy metals in soil samples were used to assess the real impact of the transposition of the different LVs. Current work aimed to introduce a methodological approach to quantify the grade of the compliance of SS directives by member states in Europe and to identify the soil samples exceeding the LVs as laid down. In practical terms, 14,726 agricultural soils from the LUCAS 2009 topsoil database were used not only to compare the concentration of each heavy metal (As, Cd, Co, Cr, Cu, Hg, Ni, Pb, and Zn) with its corresponding EU- and national-level but also taking into consideration soils factors such as texture and pH which are currently being used to lay down the reference values at national and European level, respectively. The physical and chemical properties of each soil sample of the LUCAS 2009 database were characterized based on standard sampling and analytical procedures (Tóth et al. 2013a, b). Additionally, the application of SS on agricultural soils could be readily monitored by using the subsequent LUCAS 2015 (Orgiazzi et al. 2018) and LUCAS 2018 (Fernandez-Ugalde et al. 2018; Orgiazzi et al. 2022) datasets and the upcoming LUCAS 2022 database (Ballin et al. 2022) to assess not only the grade of the compliance but also to quantify the impact of the SS disposal on agricultural land over time by using the proposed contamination indices.

### The choice of strategy

Single Contamination Rate Index (CRI) and overall Contamination Rate Index (OCRI) were used to quantify the contamination level when the concentration of each heavy metal in each LUCAS 2009 point is related to EU- and national-reference values. This methodological approach is in line with that widely used to assess the contamination of soils with trace elements by using pollution indices (Kowalska et al. 2018; Ferreira et al. 2022) as measured concentrations are compared to reference values (geochemical background levels, threshold values, toxicity levels, etc.). Additionally, the overall contamination rate index (OCRI) was determined for each LUCAS 2009 point to meet with article 5 of the SSD (Directive 86/278/EEC), as member states should prohibit the use of SS where the concentration of one or more heavy metals in the soil exceeds the LVs laid down in the corresponding Annex IA of the SSD. Additionally, the use of complex pollutant indices allows for the identification of multi-polluted soil samples because metals and metalloids such as As, Cr (VI), nickel, cadmium, and lead have been identified to have carcinogenic effects (Briffa et al. 2020), and theses indices could be used to assess the potential impact of the accumulation of heavy metals in soils when SSs are applied on agricultural soil over time.

Regarding the concentrations of heavy metals in soils, a wide variation in LVs has already been reported at the country and regional levels (Sandy 2018). For that, three different strategies (named Strategy I, II, and III in Table 1) were managed to consider all potential approaches used by Member States to lay down the LVs for concentrations of heavy metals in soils. Large differences related to the proportion and number of sites exceeding the LVs (Figs. 1 and 2) and to the contamination rate index (Fig. 3) were compared. In this work, such differences were quantified, and the number of soils exceeding LVs ranged from 1411 (10%) to 5237 (36%), respectively, when national LVs from Strategy III were compared to the lower EU LVs from Strategy I. Additionally, the number of soil samples exceeding the LVs increased by 19% (from 16 to 19%) when LVs for As and Cr were included in the national ones, showing the importance of having them in the SSD. Any significant difference in the accounting of soil samples exceeding the LVs directly impacts identifying the agricultural lands that can receive SS and the maximum quantities of SS that can be applied. It is essential to highlight that the application of sludge in agricultural land in Europe would be limited if all member states adopted the lower European LVs (EU_LL) since 10 Member States (Ireland, Italy, Austria, Slovenia, Romania, Cyprus, Malta, Bulgaria, and Greece) would be in a warning risk level to exceed the LVs as they show overall contamination rates above 80% before sludge spread.

### Analysis per heavy metals

Further analysis was carried out for each HM tested to find which would prevent the sludge application in agricultural soils. In general terms, metals such as nickel, copper, cadmium, and chromium seem to ban the application of SS in agricultural soils, as already reported elsewhere. High concentrations of cadmium and nickel in Ireland have already been reported (Zhang et al. 2008; Fay et al. 2007), as 15% and 23% of Irish soils exceed the lower EU LVs for Cd (1 mg kg^−1^) and nickel (30 mg kg^−1^), respectively. Fay et al. (2007) suggested that high cadmium and nickel levels in Irish soils are likely due to naturally high background levels and only on rare occasions due to anthropogenic activities. Similar trends were found by Ballabio et al. (2023) when creating the map of the probability of exceeding 1 mg kg^−1^ of cadmium. The southern region of Ireland showed a chance higher than 0.54 of exceeding the threshold values. In the case of copper, areas in France and Italy showed high concentrations; the copper exceedance could be related to its use as a crop protection treatment to combat mildew in vineyards. Ballabio et al. (2018) reported the largest Cu concentrations in vineyards placed in France (91.3 mg kg^−1^) and in Italy (71.9 mg kg^−1^). Droz et al. (2021) predicted that 6.3% of the dataset is above 130 mg kg^−1^.

A recent assessment of zinc in topsoils concluded that 1% of all samples had concentrations above 167 mg kg^−1^ (Van Eynde et al 2023). The two zones with Cr concentrations above 70 mg kg^−1^ (in Central to North-Western Greece and in Piemonte and the Po plain in Northern Italy) were already reported by Tóth et al. (2016) with geology-derived Cr accumulation and with large areas involving agricultural soils. Given this considerable proportion of soil samples with high Cr concentrations, we suggest that it would be worth investigating how to include LVs for Cr in the SSD but based on further analysis to differentiate between Cr(III) and Cr(VI) since hexavalent Cr [Cr(VI)] is more toxic than trivalent (Briffa et al. 2020). A proposal was submitted by the Commission on November 18th, 1988, to adhere Cr LVs of 100 and 200 mg kg^−1^ to the SSD. Still, this initiative was finally withdrawn in 1993 (JO C/1993/228/13), and no LVs for Cr were fixed. Soils with significant concentrations of As have already been identified and reported in Europe (Tóth et al. 2016; Tarvainen et al. 2013). Still, Tóth et al. (2016) used a limit value five times lower (5 mg kg^−1^) than the one used in this research (25.5 mg kg^−1^). Although geological origin was reported as the primary source of As in soils, the fact that some sites show elevated concentrations of this carcinogenic metal (Mass 1992), further research is needed in order to find a reliable and risk-based limit value for As that can be included in the SSD. Mercury shows a relatively low contamination rate for all tested countries, in line with previous findings (Panagos et al. 2021; Ballabio et al. 2021), as the average topsoil Hg concentration of all LUCAS samples was 0.055 mg kg^−1^. A total of 99% of all LUCAS soil samples have Hg concentrations lower than 0.43 mg kg^−1^.

### Analysis per country

Conversely, Strategy II was applied based on soil-specific LVs, as some member states such as Bulgaria, Malta, Portugal, Slovenia, Spain, and the Carinthia region (Austria) fixed the LVs according to soil pH values (Strategy II in Table 1). Including soil pH to establish LVs is scientifically sound since increased pH reduces metal availability in the following order: Cd > Zn > Pb (Kicińska et al. 2022, Lindsay 1979). Most stringent EU-LVs were laid down for acid soils (pH < 6), and member states were allowed to exceed the LVs laid down for soils with pH between 6 and 7 by 50% when pH values are significantly higher than 7. This extension was initially defined for three metals (zinc, nickel, and copper). Still, some member states such as Portugal, Malta, Spain, and Carinthia extended this criterion to the other metals (Cd, Hg, and Pb). When soil pH values were analyzed from LUCAS 2009 agricultural soils, four Member States (Sweden, Finland, Poland, and Luxembourg) showed average pH values below 6. It would indicate that these Member States should adopt the strictest EU LVs from SSD. All national LVs from Sweden were equal to or lower than those from lower Europe. In the case of Finland, the LVs of cooper (100 mg kg^−1^), nickel (60 mg kg^−1^), and lead (60 mg kg^−1^) should be revised, and the lower ones (50, 30, and 50 mg kg^−1^, respectively) should be transposed as large solubilization rate in soils is expected. No in-depth discussion can be performed for Luxembourg as only one soil sample was analyzed, but, taking into consideration the soil pH (5.6), all LVs should be revised in order to adopt the lower ones. The national LVs in Poland were fixed by using the soil texture instead of soil pH as a soil indicator. Thus, lower LVs were laid down for sandy soils (light soils), and conversely, the upper LVs corresponded to the clayey soils (heavy soils). No significant soil samples exceeding LVs in Poland were identified since contamination rates showed the lowest values (Figs. 3 and 4), but either the lower EU LVs or the LVs fixed for light soils should be laid down in accordance with the average soil pH. The large variety among LVs can be partly explained by the fact that these LVs are based on aqua regia soil destructions, which are measurements of the pseudo-total concentrations. These concentrations are not directly linked to metal availability and thus possible (eco-)toxicological risks (Chapman et al. 2013; Nolan et al. 2003). The large variety among countries can, therefore, potentially reflect the large variety in soil properties that determine the eco-toxicological risk of pseudo-total heavy metal concentrations. However, the variety within countries of these soil properties is already quite large (Ballabio et al. 2016; 2019). Studies have indeed shown that these soil properties do affect the availability of pseudo-total metal concentrations in soils towards plants and organisms (De Vries et al. 2011; Tipping et al. 2003; Groenenberg et al. 2010) and the associated risk (Nolan et al. 2003). Our results have clearly shown that it does matter which LVs are used to assess HM contamination in soils. We cannot assess whether one of the sets of LVs for HM concentration in soils is most related to the actual risk of HM in soils, as they should be compared with other soil screening values to determine the impact of these LVs on human health. Carlon et al. (2007) reported soil screening values as generic quality standards to match soil pollution and human health. They were classified based on the risk levels (negligible, intermediate/warning, and potentially unacceptable). Values below negligible risk could correspond to threshold levels, and they would be related to natural contamination. The other ones, intermediate and potentially unacceptable risks, used to be related to anthropogenic practices. Lower and upper LVs of concentrations of heavy metals in soils as laid down in SS directive (Table 1) can be compared to those median values (mg kg^−1^) for negligible-intermediate-potentially unacceptable levels as reported by Carlon et al. (2007) that are 29–30-50 (As), 0.7–3.0–6.0 (Cd), 115–250-275 (Cr), 36–110-345 (Cu), 0.3–2.5–10 (Hg), 35–140-175 (Ni), 83–195-450 (Pb), and 140–500-700 (Zn), respectively. Maximum LVs of concentrations for cadmium (1.0–3.0), mercury (1.0–1.5), nickel (30–75), and Zn (150–300) fall between negligible and intermediate risk levels. The lower limit value for Pb (50) is below a negligible level. Upper LVs for Pb (300) and Cu (140) would be above the intermediate risk level. LVs above intermediate risk level should be revised as significant and harmful effects on human health could be reported as they are exceeded. Limit values for As (25.5) and Cr (100) were used as average values from those national legislations with available data as they are not reported in the current Sewage Sludge European Legislation. Both are below intermediate risk levels, so they could be proposed to be used as LVs. The distribution of soil samples exceeding LVs by As is in line with those reported by Clarire-Froger et al. (2023) for France, as a significant number of LUCAS 2009 points with a concentration of As higher than 25 mg kg^−1^ were found As. They reported that 25 mg kg^−1^ of As could be used as a threshold value to further investigate the As bioavailability fraction. However, other more stringent LVs for As (5 mg kg^−1^) were reported by the Ministry of the Environment of Finland (MEF 2007), and further analysis should be conducted to include LVs for As in Sewage Sludge legislation as it is a naturally occurring ubiquitous metalloid and a Class I human Carcinogen (Briffa et al. 2020). Therefore, our results clearly show that it does matter which limits are used for assessing HM contamination rates and the potential impact of SS application on agricultural soils.

## Conclusions

The first exploratory quantitative analysis of the compliance grade of the SS Directive in Europe has been performed in this work by using the concentrations of heavy metals (As, Cd, Cu, Cr, Hg, Ni, Pb, and Zn) from the 14,726 agricultural soil samples of LUCAS 2009 topsoil database as actual starting point.

The use of a single contamination rate index (CRI) and integrated Contamination Index (OCRI) not only identifies the agricultural soils exceeding the LVs and no sludge could be applied but also quantifies the grade of contamination to predict warning agricultural soils where further analysis and monitoring activities should be implemented before application of sludges. In total, 10%, 36%, and 19% of the LUCAS 2009 agricultural soil samples exceeded the LVs when EU_UL, EU_LL from strategy I and national ones from strategy III were used, respectively. Analysis of the proportion of soil samples exceeding the LVs allows us to conclude that the EU LVs for concentrations of heavy metals in soils were transposed separately by the Member States, but anyway, national LVs fall into the EU LVs interval indicating that all of them are complying with the SSD LVs when separately analyzed. The number of soil samples exceeding LVs increased by 19% when data from As and Cr were taken into consideration as laid down by member states. Limit values for concentrations of As and Cr should be included in the Sewage Sludge directive due to its impact on human health. As laid down in the SS Directive, most of the LVs for the concentration of HMs in soils fall between negligible and intermediate risk levels when generic quality standards between soil pollution and human health were compared. Further analysis should be conducted in order to assess the potential ecological risk of heavy metals from the application of SS on agricultural soil in Europe.

Using the soil pH as a criterion to fix the LVs, it concluded that 12% and 16% of agricultural soils exceeded the LVs when EU and national ones were compared. In general terms, all member states show similar or stricter LVs than those laid down in the SSD. Member States (Finland, Poland, and Luxembourg) with acid soils could adopt more stringent LVs to avoid the potential harm of the increased solubilization of heavy metals in soils. Our work shows quantitatively that different inventories of soil samples exceeding LVs will significantly affect sludge disposal and monitoring, remediation and restoration actions. If all member states adopt the lower European LVs (EU_LL), as currently laid down in the SSD, one-third of European agricultural land exceeds the LVs for at least one HM, and no sludges could be spread, per Article 5 of SSD. These figures decrease drastically by 64% if member states adopt the EU levels by using soil pH as a discriminant factor.

The actual soil parameters, such as heavy metal concentrations and soil pH values from the LUCAS 2009 database, could be used by SSD-involved policy stakeholders not only to lay down the LVs for concentrations of heavy metal in soils but also for monitoring the compliance grade by using the experimental data got from other LUCAS surveys over time (past and upcoming LUCAS datasets).

## Supplementary Information

Below is the link to the electronic supplementary material.

Supplementary file1 (DOCX 1467 KB)
