# Supplementary material for: Quantitative analysis of the compliance of EU Sewage Sludge Directive by using the heavy metal concentrations from LUCAS topsoil database
Source: Environ Sci Pollut Res Int. 2024 Jan 16;32(28):16554–69. doi: 10.1007/s11356-024-31835-y (PMC12325391; doi:10.1007/s11356-024-31835-y)
Supplement: Supplementary file 1 — Supplementary file1 (DOCX 1467 KB) [file 11356_2024_31835_MOESM1_ESM.docx]

**Supplementary material**

**Quantitative analysis of the compliance of EU Sewage Sludge Directive by using the heavy metal concentrations from LUCAS topsoil database.**

Felipe Yunta*, Calogero Schillaci, Panos Panagos, Elise Van Eynde, Piotr Wojda, Arwyn Jones

European Commission, Joint Research Centre (JRC), Ispra, VA, Italy

***Corresponding author.**[**felipe.yunta-mezquita@ec.europa.eu**](mailto:felipe.yunta-mezquita@ec.europa.eu)


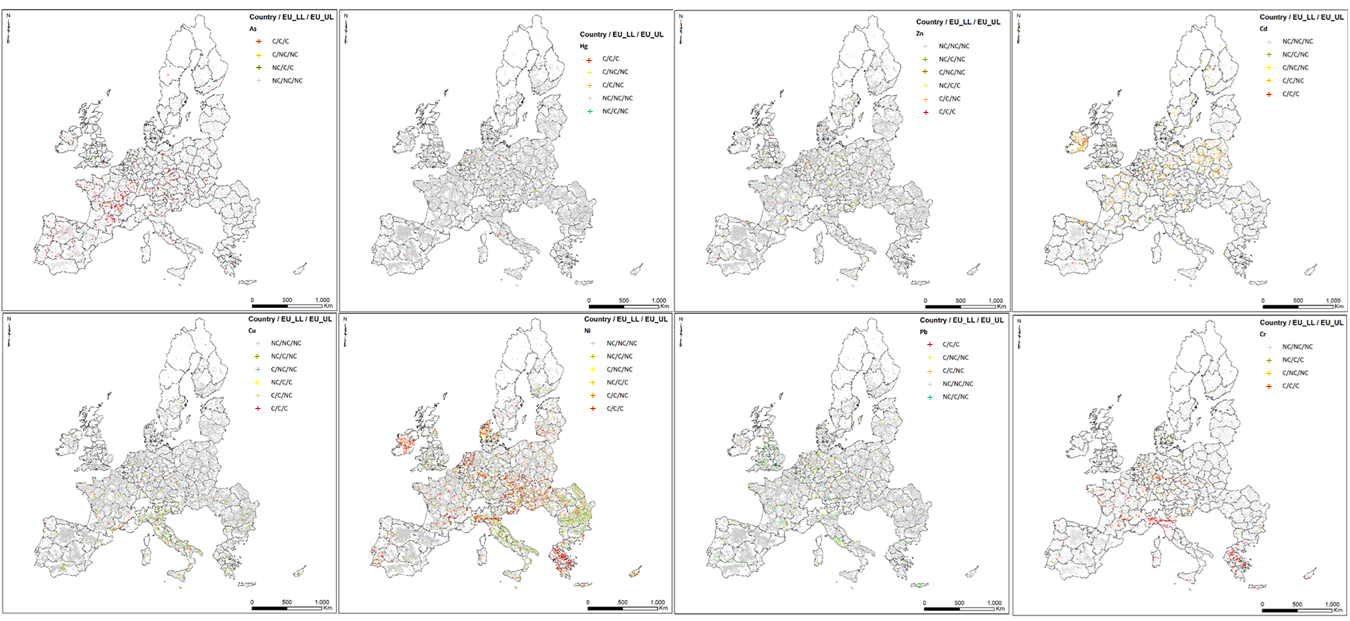


Figure 1S. Distribution of agricultural soils from LUCAS 2009 points in accordance with strategy I (Upper and Lower LVs) and strategy III (National LVs). Distribution of soil samples exceeding (Contaminated-C) and no exceeding (Non-contaminated -NC) were compared for each heavy metal (As, Cd, Cu, Cr, Hg, Ni, Pb, and Zn).


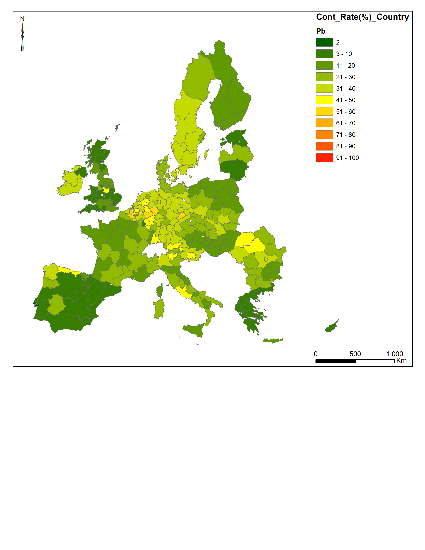

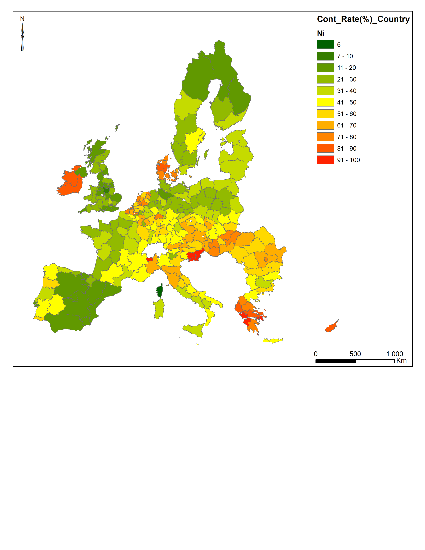

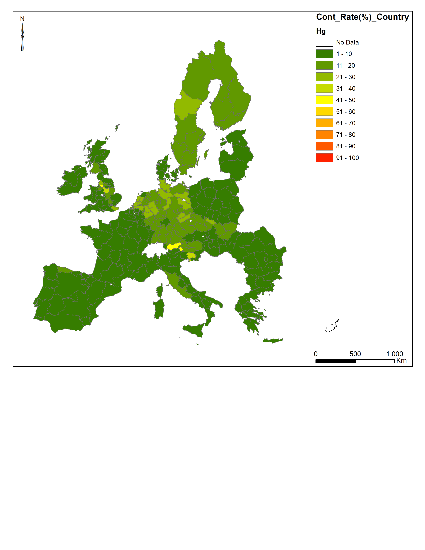

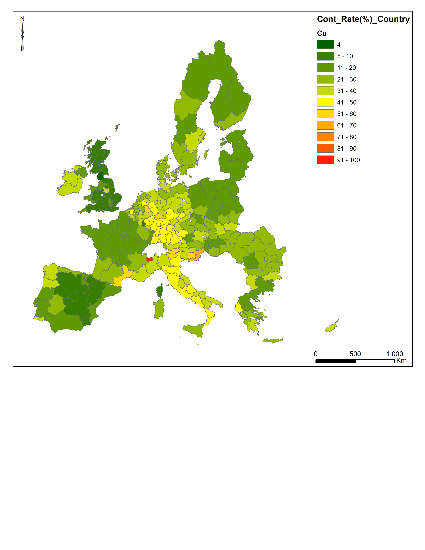

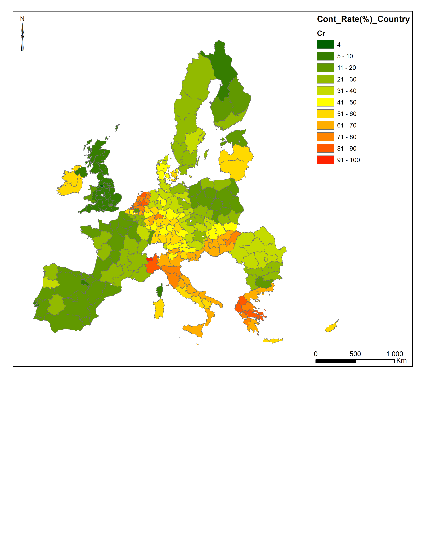

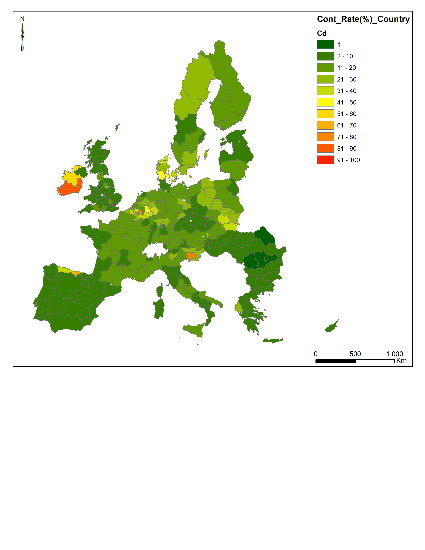

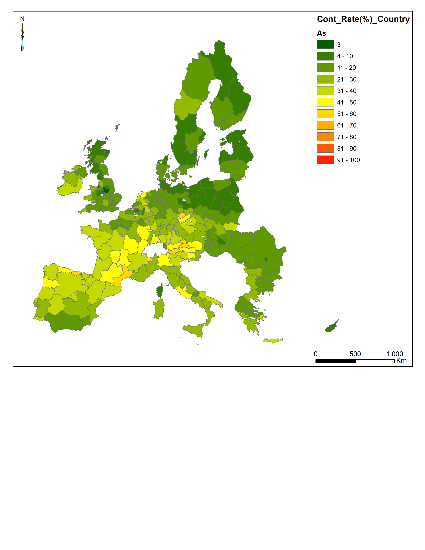

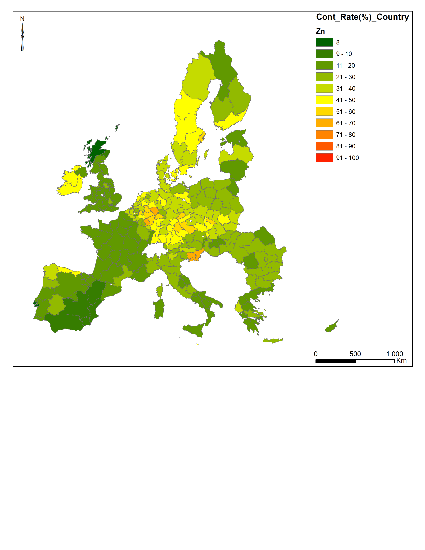


Figure 2S. Spatial distribution of CRI values for each heavy metal at NUTS2 level from LUCAS 2009 database for agricultural land (Cropland and Grassland) by using the national LVS in accordance with the strategy III.


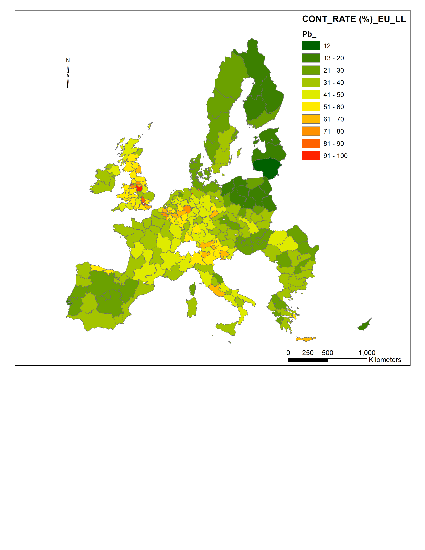

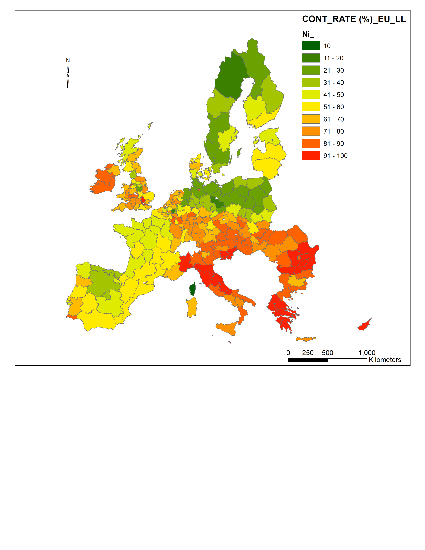

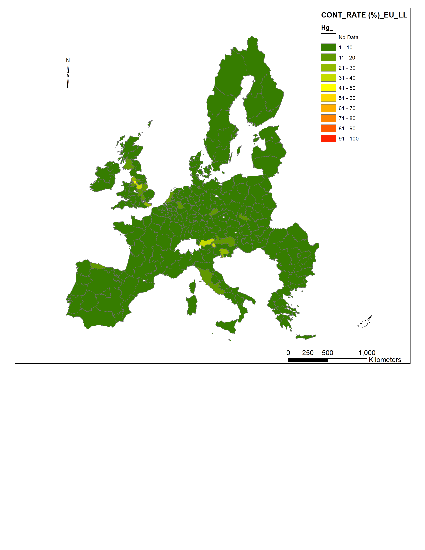

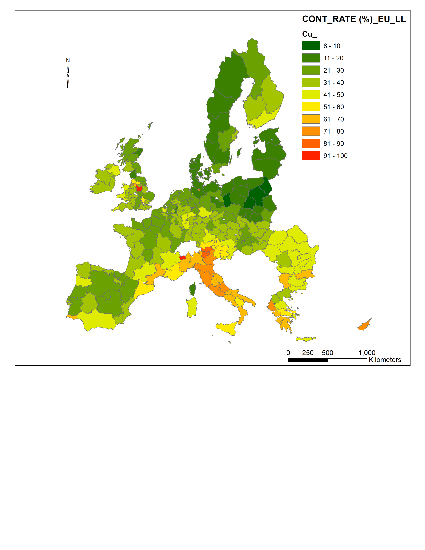

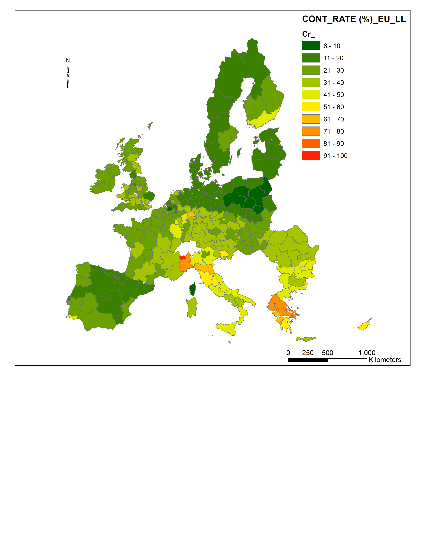

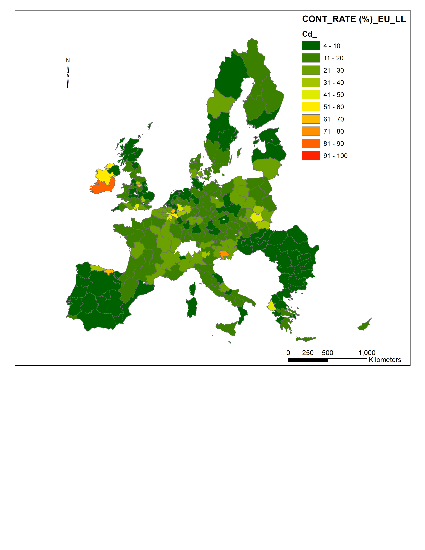

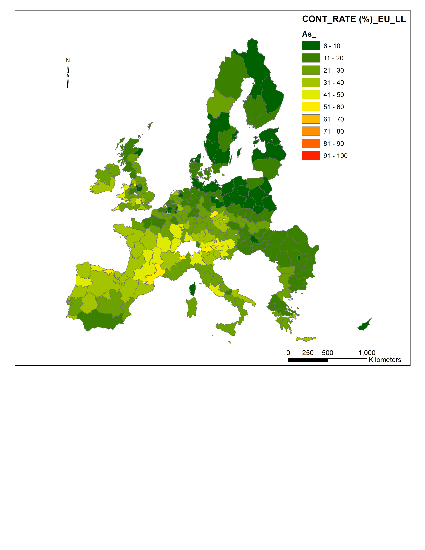

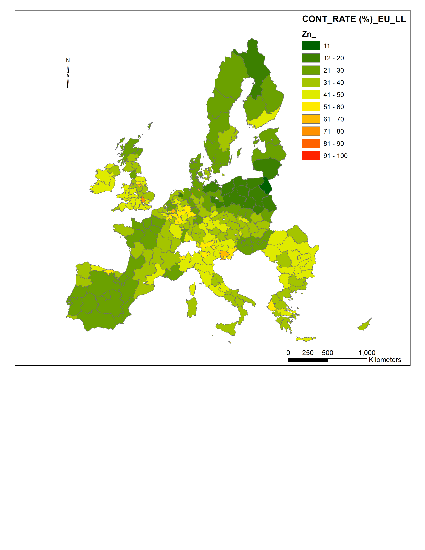


Figure 3S. Spatial distribution of CRI values for each heavy metal at NUTS2 level from LUCAS 2009 database for agricultural land (Cropland and Grassland) by representing the lower EU LVs in accordance with the strategy I.


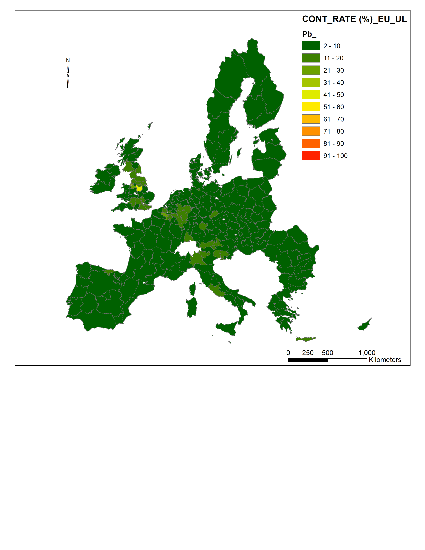

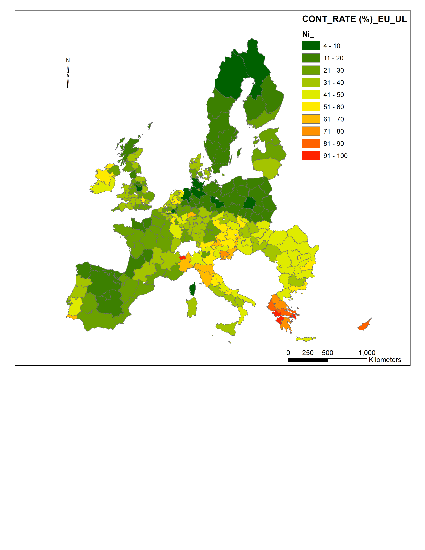

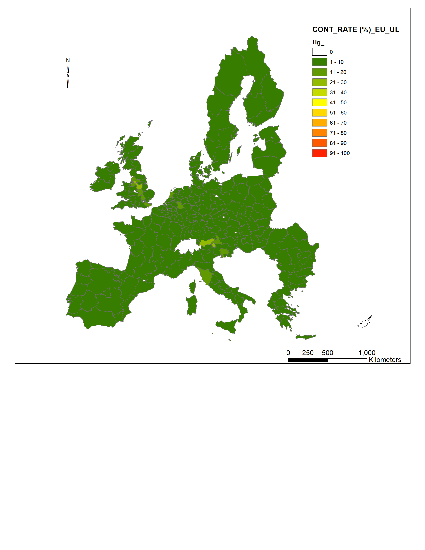

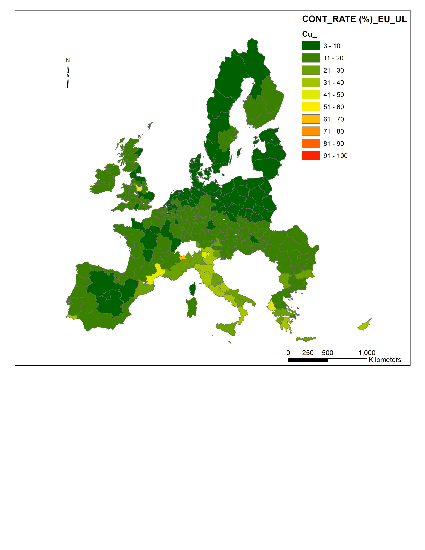

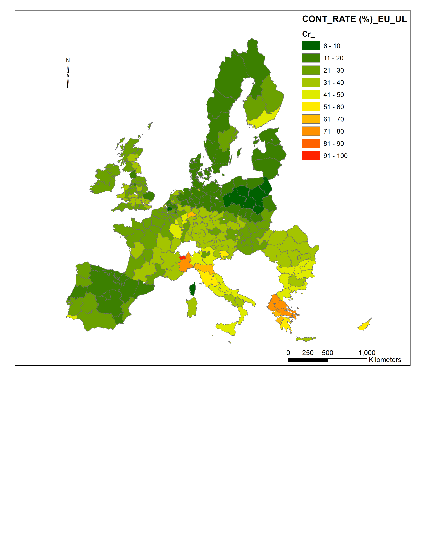

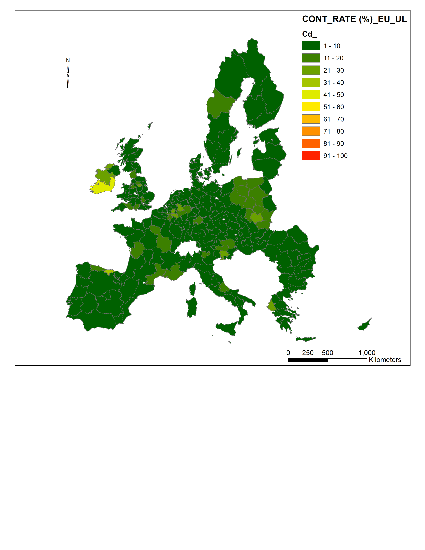

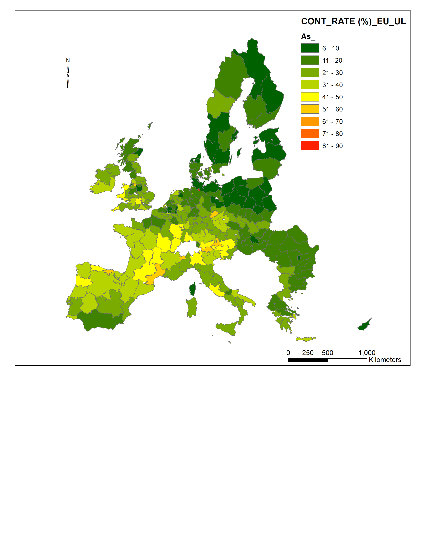

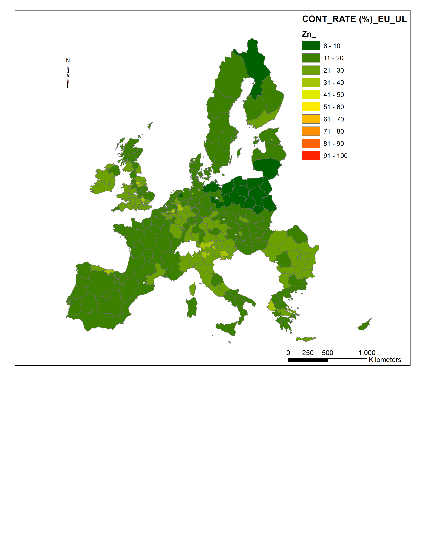


Figure 4S. Spatial distribution of CRI values for each heavy metal at NUTS2 level from LUCAS 2009 database for agricultural land (Cropland and Grassland) by representing the upper EU LVs in accordance with the strategy I.


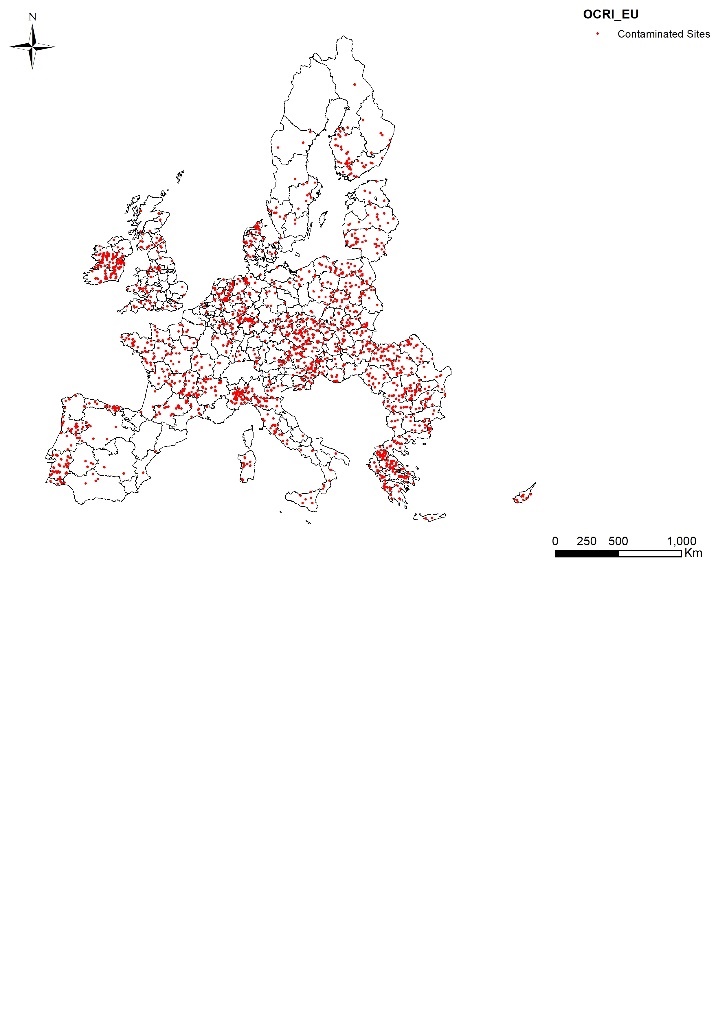

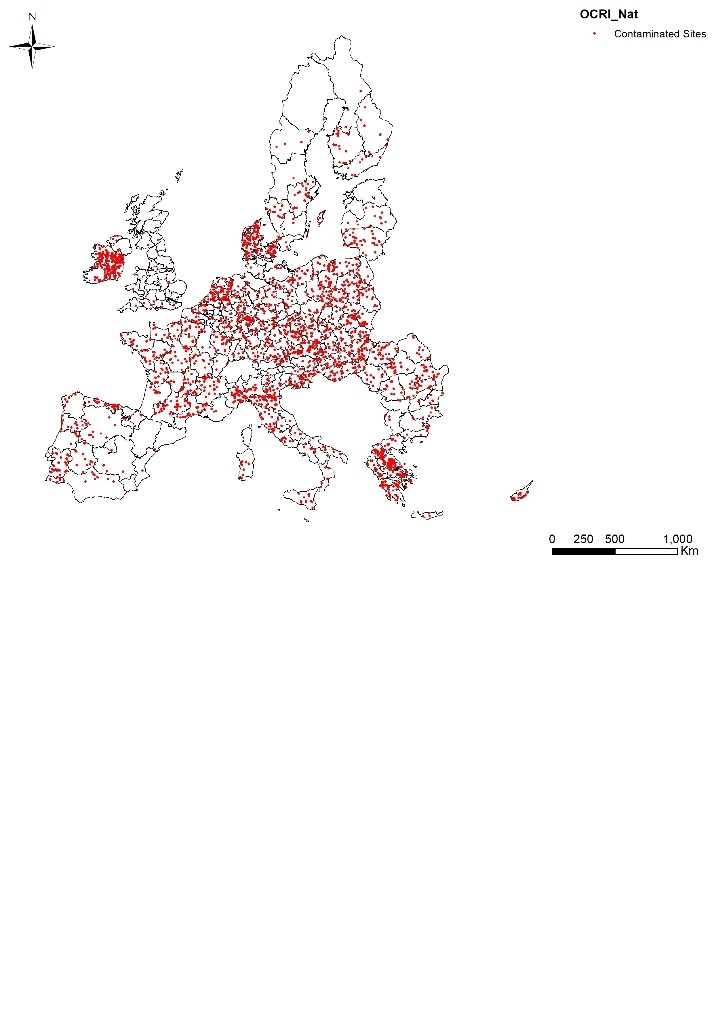


Figure 5S. Spatial distribution of the soil samples exceeding LVs for agricultural soils by using EU-levels (left) and National levels (right) as defined in the strategy II and strategy III respectively. Sites were labelled as contaminated when OCRI ≥ 100 for each LUCAS 2009 point.

Table 1S. Arithmetic average of single contaminated rate indices (CRI) at NUTS0 level (country) by using the LVs (Table 1) defined in Strategy II (OCRI_EU) and Strategy III (OCRI_Nat). Standard deviations are shown in parentheses.

|  |  | CRI_EU | | | | | | | | | | | | |  | | CRI_NAT | | | | | | | | | | | |
| --- | --- | --- | --- | --- | --- | --- | --- | --- | --- | --- | --- | --- | --- | --- | --- | --- | --- | --- | --- | --- | --- | --- | --- | --- | --- | --- | --- | --- |
| Country | ***N*** | **Cd** | | **Cu** | | **Ni** | | **Pb** | | **Zn** | | **Hg** | |  | | **Cd** | | | **Cu** | | **Ni** | | **Pb** | | **Zn** | | **Hg** | |
| Austria | *289* | 10.0 | (18.8) | 22.8 | (19.9) | 59.2 | (32.7) | 17.8 | (20.5) | 26.6 | (18.7) | 6.4 | (10.3) |  | | 14.6 | | (24.2) | 27.2 | (17.0) | 64.3 | (30.6) | 20.9 | (15.0) | 26.4 | (13.4) | 9.6 | (16.3) |
| Belgium | *55* | 14.2 | (19.0) | 13.5 | (10.8) | 36.2 | (33.6) | 19.9 | (27.0) | 23.1 | (18.3) | 3.2 | (2.5) |  | | 24.3 | | (17.3) | 31.5 | (10.2) | 60.5 | (34.6) | 40.3 | (21.1) | 36.0 | (15.4) | 4.5 | (3.2) |
| Bulgaria | *480* | 2.4 | (4.0) | 22.0 | (18.7) | 44.4 | (24.9) | 10.0 | (13.9) | 21.5 | (11.6) | 0.9 | (1.7) |  | | 2.7 | | (3.5) | 24.8 | (16.4) | 50.8 | (21.8) | 20.0 | (10.7) | 24.0 | (7.9) | 1.4 | (1.9) |
| Cyprus | *45* | 4.4 | (8.5) | 24.0 | (17.6) | 66.5 | (27.3) | 2.8 | (5.5) | 12.4 | (6.1) | 0.0 | (0.0) |  | | 6.6 | | (12.8) | 34.2 | (22.1) | 81.5 | (24.6) | 4.1 | (8.2) | 18.3 | (9.1) | 0.0 | (0.0) |
| Czechia | *334* | 6.8 | (10.9) | 15.6 | (13.9) | 51.3 | (34.0) | 14.7 | (17.7) | 22.9 | (15.2) | 3.9 | (6.0) |  | | 2.6 | | (2.9) | 24.6 | (16.6) | 57.1 | (31.1) | 27.2 | (14.5) | 45.2 | (19.8) | 16.5 | (12.0) |
| Germany | *1524* | 8.1 | (18.5) | 14.6 | (15.4) | 25.9 | (25.2) | 18.7 | (24.5) | 19.6 | (17.5) | 4.3 | (6.9) |  | | 15.6 | | (26.6) | 39.6 | (23.8) | 37.1 | (25.8) | 34.7 | (20.8) | 41.9 | (23.2) | 17.0 | (16.4) |
| Denmark | *203* | 9.6 | (11.7) | 10.7 | (9.4) | 40.2 | (34.6) | 11.6 | (16.4) | 15.9 | (14.8) | 1.9 | (1.7) |  | | 34.0 | | (27.9) | 23.6 | (11.5) | 75.0 | (31.3) | 29.9 | (22.7) | 35.8 | (24.0) | 4.8 | (3.5) |
| Estonia | *109* | 3.6 | (8.3) | 5.6 | (5.5) | 21.6 | (22.9) | 4.5 | (6.8) | 11.3 | (6.3) | 2.0 | (3.9) |  | | 2.8 | | (4.5) | 15.3 | (10.2) | 31.5 | (24.6) | 9.6 | (4.9) | 12.5 | (6.4) | 1.9 | (2.8) |
| Spain | *2149* | 3.5 | (11.3) | 9.1 | (11.1) | 15.9 | (13.5) | 5.9 | (10.4) | 10.5 | (10.1) | 2.1 | (6.5) |  | | 4.8 | | (12.7) | 10.9 | (13.7) | 18.6 | (18.2) | 10.2 | (14.6) | 12.0 | (12.6) | 2.9 | (8.3) |
| Finland | *413* | 9.9 | (22.5) | 28.6 | (22.3) | 36.4 | (28.4) | 14.5 | (14.6) | 26.3 | (21.4) | 2.8 | (5.4) |  | | 16.3 | | (25.3) | 18.1 | (12.7) | 24.0 | (19.4) | 16.0 | (12.2) | 31.1 | (23.4) | 14.2 | (13.6) |
| France | *2489* | 8.9 | (17.8) | 15.8 | (18.6) | 26.9 | (24.8) | 13.8 | (17.8) | 17.5 | (15.4) | 2.0 | (4.1) |  | | 11.9 | | (22.7) | 18.0 | (18.9) | 34.5 | (24.6) | 20.2 | (13.8) | 16.2 | (12.1) | 3.2 | (5.9) |
| Greece | *352* | 3.9 | (11.2) | 16.9 | (15.8) | 65.1 | (34.6) | 6.1 | (9.4) | 15.1 | (10.9) | 0.9 | (3.4) |  | | 4.7 | | (12.2) | 20.5 | (16.1) | 71.4 | (33.0) | 6.0 | (6.8) | 19.0 | (10.6) | 1.2 | (3.9) |
| Hungary | *427* | 1.9 | (3.1) | 11.9 | (11.7) | 40.9 | (29.1) | 6.8 | (10.3) | 13.8 | (10.9) | 1.8 | (4.8) |  | | 5.0 | | (5.5) | 20.6 | (12.3) | 67.6 | (28.4) | 12.9 | (6.4) | 21.4 | (10.8) | 5.8 | (8.9) |
| Ireland | *185* | 47.5 | (38.2) | 24.4 | (20.5) | 65.1 | (31.8) | 18.9 | (20.8) | 30.6 | (20.5) | 7.4 | (7.7) |  | | 66.4 | | (41.8) | 38.5 | (19.2) | 85.9 | (21.2) | 34.5 | (20.8) | 44.6 | (22.9) | 9.1 | (7.7) |
| Italy | *1136* | 4.7 | (8.3) | 23.0 | (19.4) | 40.0 | (26.9) | 8.2 | (12.0) | 16.9 | (10.7) | 2.7 | (8.8) |  | | 11.5 | | (16.5) | 38.1 | (23.8) | 49.1 | (27.0) | 23.5 | (17.9) | 21.3 | (10.8) | 4.6 | (11.0) |
| Lithuania | *283* | 8.7 | (16.6) | 6.1 | (5.7) | 32.6 | (33.0) | 3.2 | (5.5) | 8.7 | (7.3) | 1.2 | (1.8) |  | | 14.5 | | (19.4) | 10.3 | (4.5) | 39.6 | (34.6) | 7.6 | (5.9) | 10.6 | (6.4) | 2.0 | (2.0) |
| Luxembourg | *1* | 14.0 | - | 26.6 | - | 57.2 | - | 44.0 | - | 51.6 | - | 2.8 | - |  | | 4.7 | | - | 9.5 | - | 22.9 | - | 7.3 | - | 25.8 | - | 1.9 | - |
| Latvia | *210* | 3.4 | (7.1) | 6.5 | (9.7) | 29.4 | (28.6) | 5.6 | (9.0) | 11.5 | (9.2) | 1.7 | (2.6) |  | | 8.1 | | (12.2) | 11.0 | (10.7) | 30.5 | (28.6) | 21.5 | (16.9) | 32.7 | (18.5) | 5.1 | (6.3) |
| Malta | *11* | 0.8 | (0.0) | 8.9 | (0.0) | 17.8 | (0.0) | 3.2 | (0.0) | 13.8 | (0.0) | 0.5 | (0.0) |  | | 2.3 | | (0.0) | 18.7 | (0.0) | 28.6 | (0.0) | 14.3 | (0.0) | 31.0 | (0.0) | 1.2 | (0.0) |
| Netherlands | *188* | 4.7 | (7.0) | 13.0 | (16.0) | 45.8 | (37.0) | 13.9 | (17.3) | 17.4 | (14.4) | 4.9 | (10.8) |  | | 17.6 | | (20.8) | 31.5 | (21.4) | 63.7 | (35.1) | 36.8 | (21.4) | 35.3 | (23.2) | 18.9 | (19.7) |
| Poland | *1314* | 15.6 | (28.5) | 8.2 | (9.7) | 21.7 | (25.1) | 13.4 | (15.1) | 13.1 | (12.6) | 2.0 | (3.1) |  | | 18.1 | | (33.2) | 16.4 | (14.0) | 31.5 | (26.8) | 21.2 | (14.0) | 26.3 | (19.8) | 2.2 | (3.6) |
| Portugal | *220* | 3.8 | (5.2) | 22.3 | (25.3) | 50.8 | (36.3) | 16.3 | (19.8) | 20.3 | (19.2) | 1.7 | (3.2) |  | | 3.1 | | (4.9) | 20.0 | (22.2) | 45.2 | (34.9) | 11.1 | (17.2) | 17.0 | (16.4) | 1.5 | (2.3) |
| Romania | *1170* | 2.5 | (7.2) | 20.3 | (14.8) | 47.6 | (26.9) | 13.7 | (16.8) | 23.9 | (13.0) | 1.7 | (2.8) |  | | 1.9 | | (7.0) | 22.5 | (12.6) | 60.7 | (23.9) | 33.2 | (14.2) | 21.5 | (8.5) | 2.5 | (4.8) |
| Sweden | *335* | 7.6 | (17.2) | 15.9 | (16.6) | 26.1 | (25.3) | 19.3 | (17.1) | 22.7 | (15.3) | 3.7 | (3.8) |  | | 19.4 | | (26.5) | 27.0 | (21.8) | 35.2 | (28.3) | 35.9 | (19.8) | 43.3 | (24.7) | 13.8 | (13.4) |
| Slovenia | *41* | 21.8 | (24.2) | 29.2 | (21.4) | 75.6 | (27.8) | 29.1 | (29.3) | 38.0 | (20.3) | 10.7 | (15.0) |  | | 39.2 | | (36.0) | 61.5 | (30.3) | 91.3 | (17.2) | 34.5 | (17.3) | 65.8 | (31.0) | 17.0 | (22.7) |
| Slovakia | *178* | 9.1 | (13.9) | 14.8 | (13.4) | 39.1 | (31.3) | 12.0 | (14.9) | 19.4 | (11.7) | 4.2 | (7.1) |  | | 21.5 | | (26.5) | 32.9 | (17.7) | 48.6 | (29.7) | 26.7 | (15.4) | 36.2 | (14.5) | 12.2 | (16.2) |
| United Kingdom | *583* | 7.3 | (12.4) | 17.1 | (17.2) | 38.0 | (27.6) | 24.2 | (27.2) | 24.7 | (17.1) | 6.1 | (9.4) |  | | 5.6 | | (11.1) | 8.3 | (6.7) | 21.2 | (16.6) | 10.3 | (10.5) | 14.2 | (9.6) | 8.4 | (11.5) |
